# Factors contributing to poor COVID-19 outcomes in diabetic patients: Findings from a single-center cohort study

**DOI:** 10.1371/journal.pone.0290946

**Published:** 2023-08-31

**Authors:** Nosayba Al-Azzam, Sayer Al-Azzam, Basheer Khassawneh, Mohammad Araydah, Reema A. Karasneh, Mamoon A. Aldeyab

**Affiliations:** 1 Department of Physiology and Biochemistry, Faculty of Medicine, Jordan University of Science and Technology, Irbid, Jordan; 2 Department of Clinical Pharmacy, Faculty of Pharmacy, Jordan University of Science and Technology, Irbid, Jordan; 3 Department of Internal Medicine, Faculty of Medicine, Jordan University of Science and Technology, Irbid, Jordan; 4 Princess Basma Teaching Hospital, Irbid, Jordan; 5 Department of Basic Medical Sciences, Faculty of Medicine, Yarmouk University, Irbid, Jordan; 6 Department of Pharmacy, School of Applied Sciences, University of Huddersfield, Huddersfield, United Kingdom; Cairo University Kasr Alainy Faculty of Medicine, EGYPT

## Abstract

Diabetes Mellitus (DM) is a frequent comorbidity in patients infected with the SARS-CoV-2 virus. The risk of developing severe or critical COVID-19 and higher mortality was observed to be increased in diabetic patients hospitalized due to COVID-19. In this study we aimed to find out the impact of clinical characteristics, comorbidities, laboratory results, and complications on the outcomes of diabetic patients hospitalized due to COVID-19. This article is a retrospective cohort study that include diabetic patients hospitalized with COVID-19 infection. A definition of diabetes was based on the past history of diabetes or if the HbA1c was 6.5% or higher. Demographics, clinical characteristics, comorbidities, laboratory results, and complications were extracted from the electronic medical records. The mortality rate increased with increasing age (from 5.56% in younger patients to 46% in the elderly) and with severity (from 25.71% in moderate cases to 43.77% in critical cases). We found that a critical severity on admission (OR: 5.26, 95% CI: 1.28–21.66, *p* = 0.0214), a history of stroke (OR: 8.37, 95% CI: 2.2–31.88, *p* = 0.0018), and low calcium levels on admission (OR: 2.23, 95% CI: 1.01–4.91, *p* = 0.0475) were significant risk factors predicting higher COVID-19 mortality in diabetic patients. The findings of this study suggest that reduced calcium levels could potentially indicate higher mortality due to COVID-19 in patients with DM. Furthermore, careful monitoring of diabetic patients hospitalized due to COVID-19 infection, especially those with critical disease severity or those with a history of stroke, may improve their outcome and lessen mortality.

## Introduction

The novel, extremely contagious severe acute respiratory syndrome coronavirus 2 (SARS-CoV-2), which causes the coronavirus disease 2019 (COVID-19), can infect the body through binding with angiotensin-converting enzyme 2 (ACE-2) receptors at the surface of various tissue organs [1, 2]. COVID-19 was declared a global pandemic with an extremely fast contagious course on March 11, 2020, by the World Health Organization (WHO) [3]. This pandemic exerted a disastrous impact on the human race, especially on the elderly and those with multiple comorbid conditions [4]. Many variables were demonstrated to be associated with worsening outcomes and consequence for COVID-19; such as older age (> 75 years old), higher Body Mass Index (BMI), chronic kidney disease, low oxygen saturation at admission (< 92%), hypernatremia, severe lymphopenia, diabetes, and others [5, 6].

Multiple studies demonstrated that diabetes mellitus (DM) is a common comorbidity that occurs in hospitalized patients due to COVID-19, with an incidence rate of up to 58% [7, 8]. The growing chance of having a severe disease or even death among diabetic patients was observed in preceding viral epidemics such as SARS-CoV-1, influenza A (H1N1), and Middle East Respiratory Syndrome (MERS) coronavirus outbreaks [9, 10]. Likewise, the risk of severe outcomes and poor prognosis was reported to be higher in patients with DM early in the COVID-19 pandemic in China [2]. After that, studies confirmed the increased Intensive Care Unit (ICU) admission rates and mortality among diabetic patients hospitalized due to COVID-19 [11, 12].

It was evident that hyperglycemia is common in patients with COVID-19 [13]. In one study, most patients with COVID-19 had elevated fasting blood sugar (FBS) levels at admission, with a mean of 179.9 mg/dl [14]. Diabetic patients or patients with uncontrolled hyperglycemia are at a higher risk of developing a severe or critical COVID-19 due to the dysfunction of their innate immune system [15]. Therefore, strict blood glucose control in diabetic patients admitted due to COVID-19 should be the most crucial step in the management of this disease, as it has been proven to strengthen the immune system [16].

The causes of significant elevations in blood glucose levels in diabetic individuals infected with SARS-CoV-2 virus can be classified into direct and indirect causes [17]. The direct causes are those related directly to the effect of the virus itself. The virus induces inflammatory responses with an accompanying increase in various inflammatory mediators, such as cytokines, which can lead to an increase in insulin resistance and poor blood glucose control [17]. Moreover, the virus can induce a pancreatic injury by targeting ACE-2 receptors in the pancreatic islet cells [17]. On the other hand, indirect causes are related to the effect of the pandemic on blood glucose management and the effect of various COVID-19 treatments on glucose hemostasis. For instance, the use of glucocorticoid therapy in COVID-19 disease alters glucose metabolism by increasing the rates of gluconeogenesis and increasing the tissue’s insulin resistance [17].

Many variables are associated with DM, especially type-2 DM, which are also predictors for a poorer COVID-19 prognosis, such as advanced age, obesity, and multiple associated comorbidities. The role of these variables in determining the consequences and outcomes of COVID-19 in diabetic patients is yet to be established. The aim of this study is to find out the impact of clinical characteristics, comorbidities, laboratory results, and complications on the outcomes of diabetic patients hospitalized due to COVID-19.

## Materials and methods

### Study design and setting

After getting the approval from the Institutional Review Board (IRB) at the Jordan University of Science and Technology (IRB number: 27/137/2021), an observational, retrospective cohort study was conducted. Diabetic patients who were admitted at the King Abdullah University Hospital (KAUH) due to a positive COVID-19 test from the beginning of the pandemic to October 2021 were included in this study. Patients with DM were selected based on their past medical history or if their HbA1c at admission was 6.5% (48 mmol/mol) or higher. Patients aged 18 years old or younger and those who were admitted for quarantine purposes were excluded. Patients were considered to have well-controlled diabetes if the HbA1c level at admission was not higher than 7%; otherwise, they were considered to have poorly controlled diabetes [18]. The study population was divided into two groups based on the end result of the admission: those who were discharged and those who died.

### Collection of research samples and data

Demographics (age, gender, BMI, and smoking status), clinical characteristics (severity score at admission, hospital stay variables, co-morbidities, and complications), laboratory parameters, management, and clinical outcomes for each patient were extracted from the electronic medical records of the KAUH database. The severity of COVID-19 was classified according to the COVID-19 treatment guidelines by the National Institutes of Health (NIH) [19]. Laboratory tests were categorized into high, low, or normal based on the normal range of the hospital, and these ranges are available in (S1 Table). In accordance with ethical guidelines, the authors did not have access to any information that could identify individual participants during or after data collection. All data were anonymized and stored securely to ensure participant confidentiality.

### Statistical analysis

Frequencies and percentages were used to describe categorical variables. Means and standard deviations (SD) were used to describe the distribution of continuous variables. Independent group *t-test* was used to compare means for continuous variables when the data were normally distributed; otherwise, the Mann-Whitney test was used. Fisher’s exact test and the chi-square test were used to compare the proportions of categorical variables between patients who died and those who remained alive. Significant variables from the chi-square test were included in a nominal logistic regression model to evaluate their impact on the mortality of COVID-19 in diabetic patients. Kaplan-Meier survival curves and log-rank tests were used to compare the survival between the two groups. A *p-*value less than 0.05 was considered statistically significant. All analyses and graphs were generated using SPSS software and GraphPad Prism version 9.4.1.

### Ethics approval statement

This study involves human participants’ data and was approved by the Institutional Review Board (IRB) at the Jordan University of Science and Technology (IRB number: 27/137/2021). Ethical considerations were adhered to throughout the study, and appropriate participant consent was obtained. According to the regulation of KAUH, all patients treated at this facility were informed that their data may be used potentially in the conduction of clinical studies. Based on that, patients who approved wrote an informed consent and those patients were included in this study.

## Results

### Study population and clinical characteristics

In total, 606 patients diagnosed with DM and admitted to KAUH due to COVID-19 were included in this study (Table 1). The mean age was 64.71 years old, with most of the patients being in the older age category (>65 years old), and 54.79% of the study population were males. Most patients were obese (46.04%) or overweight (32.18%). A total of 449 (74.09%) patients were non-smokers, while 66 (10.89%) and 91 (15.02%) were active smokers and ex-smokers, respectively. Based on the severity of COVID-19, patients were classified into the following categories: moderate (11.55%), severe (34.16%), and critical (54.29%). Approximately 66% of the patients had poorly controlled diabetes (HbA1c > 7.0%), whereas 34% had well-controlled diabetes. The most common comorbidities were hypertension and IHD, representing 68.65% and 23.1% of the study population, respectively. In the present study, 223 patients (36.8%) were dead and 383 patients (63.2%) were discharged alive.

**Table 1:** Demographics and clinical characteristic of hospitalized DM patients with COVID-19 by the end-result of admission.

Variable	Total (n = 606) n (%total)	Death (n = 223) n (% row)	Discharged (n = 383) n (% row)	*p* value
**Age**				**< 0.0001**
19–40	18 (2.97%)	1 (5.56%)	17 (94.44%)	
41–65	288 (47.52%)	84 (29.17%)	204 (70.83)	
>65	300 (49.5%)	138 (46%)	162 (54%)	
**Gender**				0.3239
Female	274 (45.21%)	95 (34.67%)	179 (65.33%)	
Male	332 (54.79%)	128 (38.55%)	204 (61.45%)	
**BMI**				0.3348
Normal	73 (12.05%)	33 (45.21%)	40 (54.79%)	
Obesity	279 (46.04%)	105 (37.63%)	174 (62.37%)	
Overweight	195 (32.18%)	69 (35.38%)	126/ (64.62%)	
**Smoking status**				0.0703
Active smoker	66 (10.89%)	25 (37.88%)	41 (62.12%)	
Ex-smoker	91 (15.02%)	43 (47.25%)	48 (52.75%)	
Non-smoker	449 (74.09%)	155 (34.52%)	294 (65.48%)	
**Severity**				**0.0005**
Moderate	70 (11.55%)	18 (25.71%)	52 (74.29%)	
Severe	207 (34.16%)	61 (29.47%)	146 (70.53%)	
Critical	329 (54.29%)	144 (43.77%)	185 (56.23%)	
**Diabetes control**				0.8833
Well-controlled	207 (34.16%)	77 (37.2%)	130 (62.8%)	
Poorly-controlled	399 (65.84%)	146 (36.59%)	253 (63.41%)	
**Comorbidities**				
Hypertension	416 (68.65%)	167 (40.14%)	249 (59.86%)	**0.0115**
Dyslipidemia	45 (7.43%)	13 (28.89%)	32 (71.11%)	0.2528
IHD	140 (23.1%)	60 (42.86%)	80 (57.14%)	0.0901
Atrial fibrillation	23 (3.8%)	9 (39.13%)	14 (60.87%)	0.8131
Heart failure	63 (10.4%)	35 (55.56%)	28 (44.44%)	**0.0011**
Asthma	27 (4.46%)	10 (37.04%)	17 (62.96%)	0.979
COPD	5 (0.83%)	3 (60%)	2/ (40%)	0.28
OSA	5 (0.83%)	3 (60%)	2 (40%)	0.28
Stroke	56 (9.24%)	29 (51.79%)	27 (48.21%)	**0.0146**
CKD	61 (10.07%)	40 (65.57%)	21 (34.43%)	**< 0.0001**
ESRD	8 (1.32%)	4 (50%)	4 (50%)	0.438
Liver Cirrhosis	1 (0.17%)	1 (100%)	0 (0%)	0.1897
Hypothyroidism	33 (5.45%)	16 (48.48%)	17 (51.52%)	0.1523
Gout	32 (5.28%)	11 (34.38%)	21 (65.63%)	0.7702
Rheumatoid arthritis	11 (1.82%)	6 (54.55%)	5 (45.45%)	0.2181
Thromboembolic event	11 (1.82%)	6 (54.55%)	5 (45.45%)	0.2181
Immunocompromised	18 (2.97%)	10 (55.56%)	8 (44.44%)	0.0939
Malignancy	30 (4.95%)	15 (50%)	15 (50%)	0.1241
BPH	35 (5.78%)	17 (48.57%)	18 (51.43%)	0.198
** Emphysema**	15 (2.48%)	13 (86.67%)	2 (13.33%)	**< 0.0001**

**IHD**: Ischemic Heart Disease, **COPD**: Chronic Obstructive Pulmonary Disease, **OSA**: Obstructive Sleep Apnea, **CKD**: Chronic Kidney Disease, **ESRD**: End Stage Renal Disease, and **BPH**: Benign Prostatic Hyperplasia.

### Outcomes for diabetic patients hospitalized due to COVID-19

Out of the 606 diabetic patients who were involved in this study, 223 (36.8%) were deceased, and 383 (63.2%) were discharged (Table 1). Patients who deceased and those who were discharged were statistically different in terms of age and disease severity on admission (*p*< 0.0001 and *p* = 0.0005, respectively). Among the classified age groups, the highest percentage of deaths was among patients who were older than 65 years old (46%), and the patients aged 19–40 years old (5.56%) had the lowest percentages of death. Gender, BMI, and smoking status were not significant factors for mortality. According to the severity classification groups, the highest percentage of deaths was among the critical cases (43.77%), while the lowest percentage of deaths was in the moderate cases (25.71%).

### The impact of associated comorbidities on the death rate in diabetic patients hospitalized due to COVID-19

**Fig 1:**
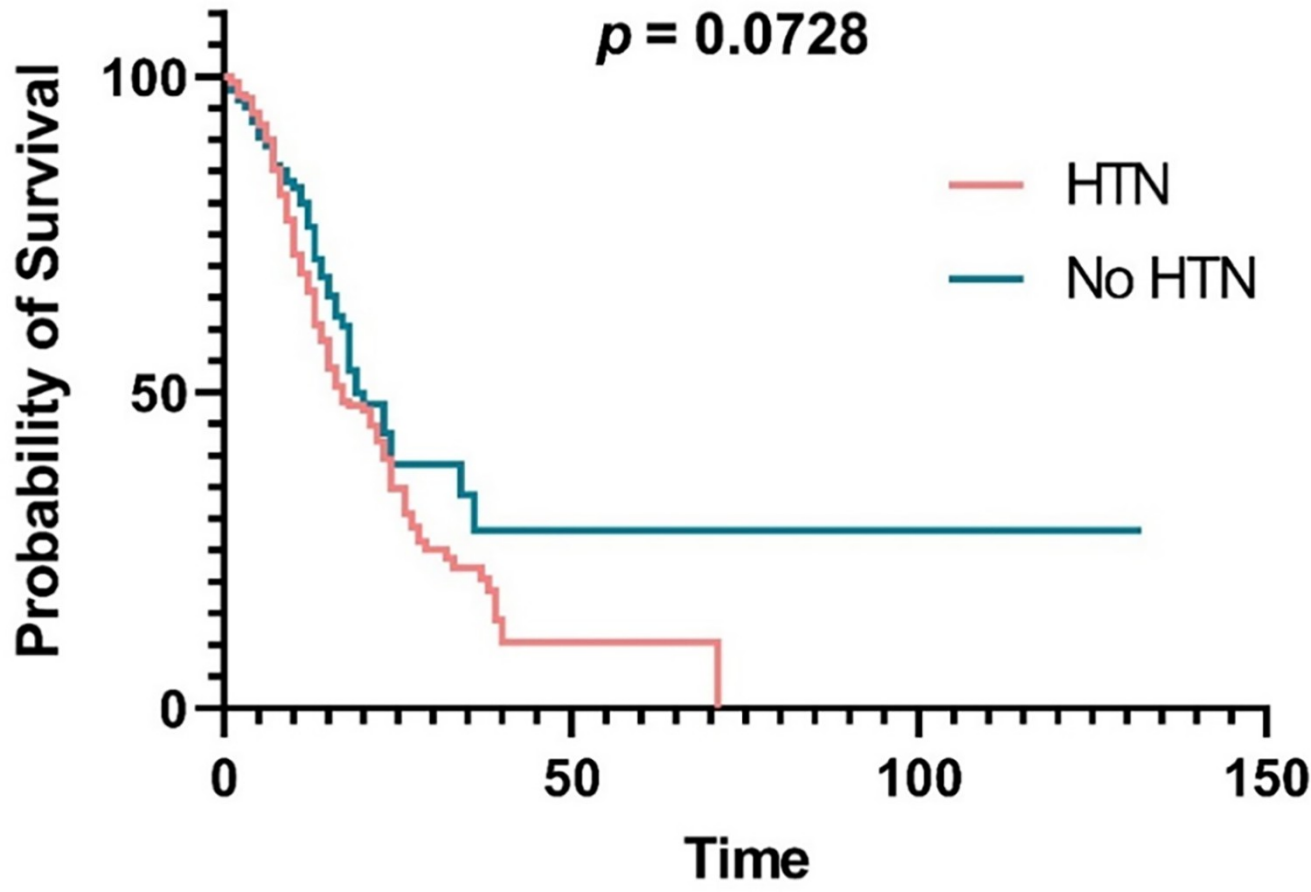
Kaplan-Meier graph comparing the survival probability between diabetic patients with and without hypertension (HTN) hospitalized due to COVID-19.

Among comorbidities, the mortality rate was significantly higher than the discharge rate in patients who had a history of heart failure (55.56% vs. 44.44%, *p* = 0.0011), stroke (51.79% vs. 48.21%, *p* = 0.0146), CKD (65.57% vs. 34.43%, *p* <0.0001), and emphysema (86.67% vs. 13.33%, *p* < 0.0001). Surprisingly, the discharge rate was significantly higher than the mortality rate in patients with hypertension (59.86% vs. 40.14%, *p* = 0.0115) (Table 1). However, around 70% of the enrolled patients were also hypertensive. In addition, the median overall survival was 19 days for patients without hypertension and 17 days for patients with hypertension. Moreover, the probability of survival between diabetic patients with and without hypertension was not significantly different (*p* = 0.0728) according to the Kaplan-Meier curve and log-rank test (Fig 1).

### The impact of laboratory results on the death rate in diabetic patients hospitalized due to COVID-19

The levels of calcium, magnesium, sodium, urea, creatinine, total protein, albumin, total bilirubin, direct bilirubin, alkaline phosphatase (ALP), aspartate aminotransferase (AST), international normalized ratio (INR), creatine kinase (CK), creatine kinase MB (CK-MB), white blood cells (WBCs), red blood cells (RBCs), hemoglobin, hematocrit, red cell distribution width (RDW), lymphocytes, monocytes, and basophils were different between the dead and survived groups with a statistically significant *p*-value (Table 2). Data regarding the insignificant laboratory results are available in (S2 Table).

**Table 2:** Significant laboratory tests at admission among DM patients with COVID-19.

Variable	Total n (%total)	Death (n = 223) n (% row)	Discharged (n = 383) n (% row)	*p* value
**Calcium**				**<0.0001**
High	1 (0.19%)	1 (100%)	0 (0%)	
Low	181 (34.81%)	95 (52.49%)	86 (47.51%)	
Normal	338 (65%)	104 (30.77%)	234 (69.23%)	
**Magnesium**				**0.0219**
High	16 (3.12%)	10 (62.5%)	6/16 (37.5%)	
Low	303 (59.06%)	106 (34.98%)	197 (65.02%)	
Normal	194 (37.82%)	85 (43.81%)	109 (56.19%)	
**Sodium**				**0.0004**
High	12 (2.09%)	11 (91.67%)	1 (8.33%)	
Low	235 (41.01%)	87 (37.02%)	148 (62.98%)	
Normal	326 (56.89%)	116 (35.58%)	210 (64.42%)	
**Urea**				**<0.0001**
High	245 (42.83%)	123 (50.2%)	122 (49.8%)	
Low	5 (0.87%)	1 (20%)	4 (80%)	
Normal	322 (56.29%)	90 (27.95%)	232 (72.05%)	
**Creatinine**				**<0.0001**
High	204 (35.6%)	106 (51.96%)	98 (48.04%)	
Low	64 (11.17%)	19 (29.69%)	45 (70.31%)	
Normal	305 (53.23%)	89 (29.18%)	216 (70.82%)	
**Total protein**				**<0.0001**
High	6 (1.09%)	0(0%)	(100%)	
Low	81 (14.67%)	51 (62.96%)	30 (37.04%)	
Normal	465 (84.24%)	160 (34.41%)	305 (65.59%)	
**Albumin**				**<0.0001**
Low	288 (52.84%)	135 (46.88%)	153 (53.13%)	
Normal	257 (47.16%)	73 (28.4%)	184 (71.6%)	
**Total bilirubin**				**0.0066**
High	16 (2.88%)	12 (75%)	4 (25%)	
Low	1 (0.18%)	0 (0%)	1 (100%)	
Normal	538 (96.94%)	200 (37.17%)	338 (62.83%)	
**Direct bilirubin**				**0.0038**
High	150 (27.03%)	72 (48%)	78 (52%)	
Normal	405 (72.97%)	140 (38.2%)	265 (65.43%)	
**ALP**				**0.0436**
High	66 (11.87%)	33 (50%)	33 (50%)	
Low	15 (2.7%)	3 (20%)	12 (80%)	
Normal	475 (85.43%)	176 (37.05%)	299 (62.95%)	
**AST**				**0.0068**
High	217 (39.1%)	98 (45.16%)	119 (54.84%)	
Normal	338 (60.9%)	114 (33.73%)	224 (66.27%)	
**INR**				**0.0008**
High	217 (47.8%)	102 (47%)	115 (53%)	
Normal	237 (52.2%)	75 (31.65%)	162 (68.35%)	
**CK**				**0.0021**
High	118 (29.35%)	57 (48.31%)	61 (51.69%)	
Low	6 (1.49%)	1 (16.67%)	5 (83.33%)	
Normal	278 (69.15%)	85 (30.58%)	193 (69.42%)	
**CK-MB**				**0.0026**
High	132 (37.71%)	63 (47.73%)	69 (52.27%)	
Normal	218 (62.29%)	69 (31.65%)	149 (68.35%)	
**WBCs**				**0.0024**
High	144 (24.7%)	71 (49.31%)	73 (50.69%)	
Low	56 (9.61%)	21 (37.5%)	35 (62.5%)	
Normal	383 (65.69%)	126 (32.9%)	257 (67.1%)	
**RBCs**				**0.0002**
High	29 (4.97%)	10 (34.48%)	19 (65.52%)	
Low	123 (21.06%)	66 (53.66%)	57 (46.34%)	
Normal	432 (73.97%)	143 (33.1%)	289 (66.9%)	
**Hemoglobin**				**0.0021**
High	9 (1.54%)	5 (55.56%)	4 (44.44%)	
Low	276 (47.26%)	122 (44.2%)	154 (55.8%)	
Normal	299 (51.2%)	92 (30.77%)	207 (69.23%)	
**Hematocrit**				**0.0308**
High	4 (0.68%)	2 (50%)	2 (50%)	
Low	373 (63.87%)	154 (41.29%)	219 (58.71%)	
Normal	207 (35.45%)	63 (30.43%)	144 (69.57%)	
**RDW**				**0.0062**
High	197 (33.73%)	89 (45.18%)	108 (54.82%)	
Normal	387 (66.27%)	130 (33.59%)	257 (66.41%)	
**Lymphocytes**				**0.0257**
High	10 (1.71%)	3 (30%)	7 (70%)	
Low	486 (83.22%)	194 (39.92%)	292 (60.08%)	
Normal	88 (15.07%)	22 (25%)	66 (75%)	
**Monocytes**				**0.0140**
High	104 (17.81%)	28 (26.92%)	76 (73.08%)	
Low	30 (5.14%)	16 (53.33%)	14 (46.67%)	
Normal	450 (77.05%)	175 (38.89%)	275 (61.11%)	
**Basophiles**				**0.0223**
High	8 (1.39%)	0 (0%)	8 (100%)	
Low	468 (81.53%)	185 (39.53%)	283 (60.47%)	
Normal	98 (17.07%)	30 (30.61%)	68 (69.39%)	

**ALP**: Alkaline Phosphatase, **AST**: Aspartate Aminotransferase, **INR**: International Normalized Ratio, **CK**: Creatinine Kinase, **CK-MB**: Creatinine Kinase-MB, **WBCs**: White Blood Cells, **RBCs**: Red Blood Cells, **RDW**: Red Cell Distribution Width

Regarding the kidney function test (KFT) and liver function test (LFT), the mortality rate was higher in patients with high levels of urea (High: 50.2% vs. Low: 20% and Normal: 27.95%), creatinine (High: 51.96% vs. Low: 29.69% and Normal: 29.18%), ALP (High: 50% vs Low: 20% and Normal: 37.05%), and AST (High: 45.16% vs. Normal: 33.73%) (Table 2). The mortality rate was higher among patients with low levels of total protein (Low: 62.96% vs. Normal: 34.41%) and albumin (Low: 46.88% vs. Normal: 28.4%) (Table 2).

### The impact of glycemic control on the death rate in diabetic patients hospitalized due to COVID-19

**Fig 2:**
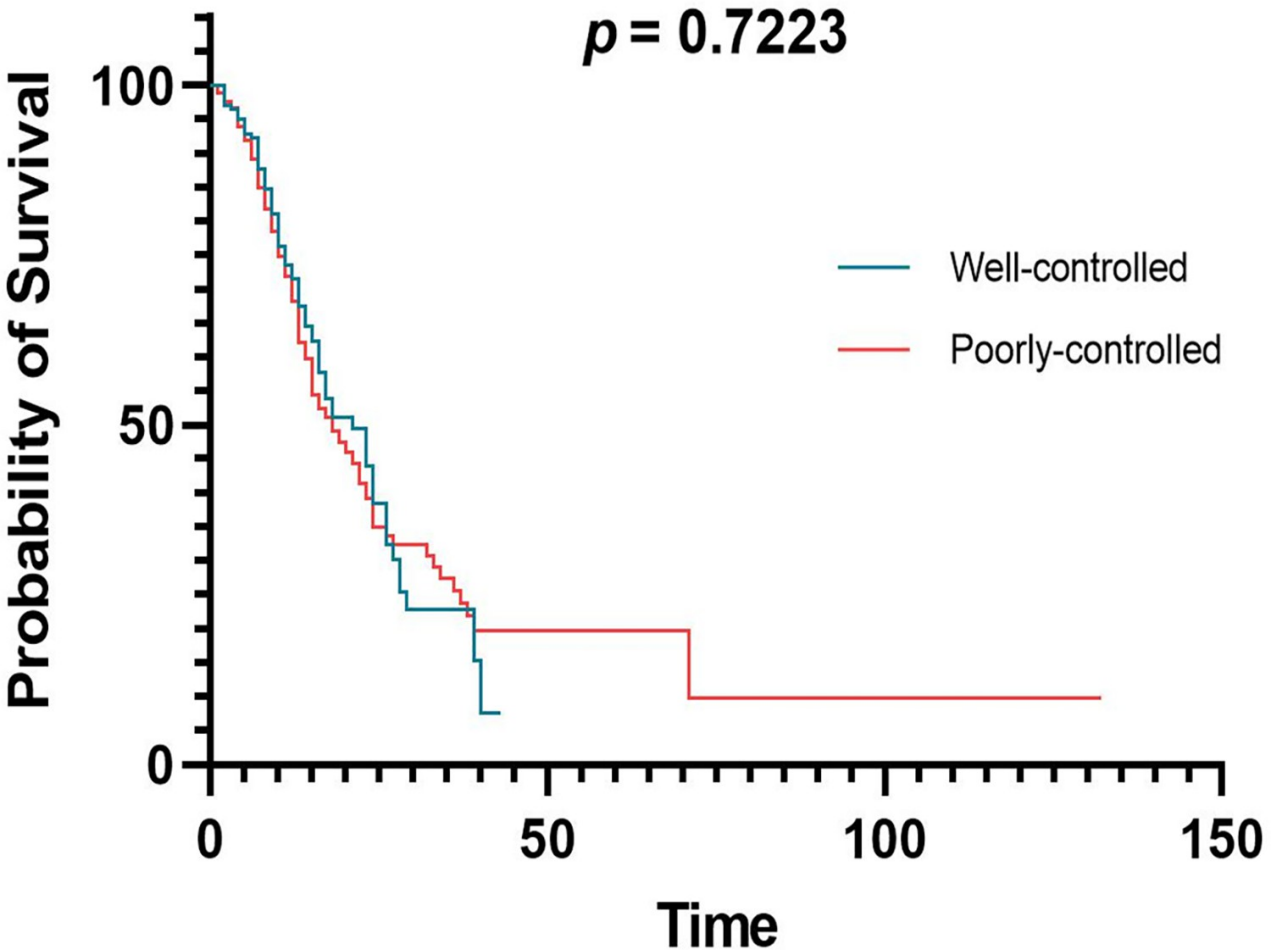
Kaplan-Meier graph for well- vs poorly-controlled diabetic patients hospitalized due to COVID-19.

To compare the mortality rate between well-controlled and poorly controlled DM patients based on HbA1c at admission, a Kaplan-Meier figure was generated, and a log-rank test was performed (Fig 2). There was no observed statistically significant difference in the survival between well- and poorly controlled DM (*p* = 0.7223).

### The impact of experienced complications on the death rate in diabetic patients hospitalized due to COVID-19

The mortality rate was significantly higher than the discharge rate in patients who were complicated with pneumothorax (88.89% vs. 11.11%, *p* < 0.0001), stroke (78.95% vs. 21.05%, *p* = 0.0001), sepsis (81.25% vs. 18.75%, *p* < 0.0001), shock (100% vs. 0%, *p* < 0.0001), acute kidney injury (AKI) (70.69% vs. 29.31%, *p* < 0.0001), arrhythmia (79.71% vs. 20.29%, p < 0.0001), and urinary tract infection (UTI) (66.67% vs. 33.33%, *p* = 0.0003) (Table 3). Also, the mortality rate was significantly higher than the discharge rate in patients who underwent hemodialysis (78.57% vs. 21.43%, *p* < 0.0001). Furthermore, the mortality was significantly higher in patients who were complicated with encephalitis (100% vs. 0%, *p* = 0.0229), acute heart failure (87.5% vs. 12.5%, p = 0.0028), and disseminated intravascular coagulopathy (DIC) (80% vs. 20%, *p* = 0.0443) during the hospital stay. Of note, the total number of patients who have experienced the aforementioned complications was less than 10.

**Table 3:** Complications occurred during hospital stay among DM patients with COVID-19.

Variable	Total n (%total)	Death (n = 223) n (% row)	Discharged (n = 383) n (% row)	*p* value
**Acute respiratory distress syndrome**	545 (89.93%)	216 (39.63%)	329 (60.37%)	**< 0.0001 **
**Acute respiratory failure**	597 (98.51%)	223 (37.35%)	374 (62.65%)	**0.0211**
**Pneumothorax**	27 (4.46%)	24 (88.89%)	3 (11.11%)	**< 0.0001**
**DVT**	10 (1.65%)	6 (60%)	4 (40%)	0.1250
**PE**	6 (0.99%)	2 (33.33%)	4 (66.67%)	0.8596
**Acute MI**	12 (1.98%)	7 (58.33%)	5 (41.67%)	0.1182
**Stroke**	19 (3.14%)	15 (78.95%)	4 (21.05%)	**0.0001**
**Sepsis**	48 (7.92%)	39 (81.25%)	9 (18.75%)	**< 0.0001**
**Shock**	24 (3.96%)	24 (100%)	0 (0%)	**< 0.0001**
**Gastrointestinal bleeding**	7 (1.16%)	4 (57.14%)	3 (42.86%)	0.2616
**Encephalitis**	3 (0.5%)	3 (100%)	0 (0%)	**0.0229**
**Acute Kidney Injury**	116 (19.14%)	82 (70.69%)	34 (29.31%)	**< 0.0001**
**Acute heart failure**	8 (1.32%)	7 (87.5%)	1 (12.5%)	**0.0028**
**Myocarditis**	2 (0.33%)	1 (50%)	1 (50%)	0.6982
**Arrhythmias**	69 (11.39%)	55 (79.71%)	14 (20.29%)	**< 0.0001**
**UTI**	33 (5.45%)	22 (66.67%)	11 (33.33%)	**0.0003**
**DIC**	5 (0.83%)	4 (80%)	1 (20%)	**0.0443**
**DKA**	30 (4.95%)	13 (43.33%)	17 (56.67%)	0.4465
**Acute pulmonary edema**	1 (0.17%)	0 (0%)	1 (100%)	0.4451
**Pleural effusion**	11 (1.82%)	4 (36.36%)	7 (63.64%)	0.9759
**Hemodialysis**	28 (4.62%)	22 (78.57%)	6 (21.43%)	**< 0.0001**

**DVT:** Deep Venous Thrombosis, **PE:** Pulmonary Embolism, **MI:** Myocardial Infarction, **UTI:** Urinary Tract Infection, **DIC:** Disseminated Intravascular Coagulopathy, and **DKA:** Diabetic Ketoacidosis

On the other hand, the mortality rate was lower with than the discharge rate with a statistical significance in patients who experienced acute respiratory failure (37.35% vs. 62.65%, *p* = 0.0211) and acute respiratory distress syndrome (39.63% vs 60.37%, *p* < 0.0001).

### Factors associated with death in diabetic patients hospitalized due to COVID-19

A nominal logistic regression analysis was performed to assess the factors associated with death among diabetic patients with COVID-19 (Table 4). We found that a critical severity on admission (OR: 5.26, 95% CI: 1.28–21.66, *p* = 0.0214), a history of stroke (OR: 8.37, 95% CI: 2.2–31.88, *p* = 0.0018), and low calcium levels on admission (OR: 2.23, 95% CI: 1.01–4.91, *p* = 0.0475) were significant risk factors predicting higher COVID-19 mortality in diabetic patients.

**Table 4:** Nominal logistic regression analysis for variable associated with mortality.

Variable	OR	Lower 95% CI	Upper 95% CI	*p* value
**Severity on admission**				
Severe vs. Moderate	3.30	0.74	14.84	0.119
Critical vs. Moderate	5.26	1.28	21.66	**0.0214**
**Comorbidities**				
Hypertension	0.60	0.26	1.38	0.225
Heart failure	0.95	0.32	2.78	0.927
Stroke	8.37	2.20	31.88	**0.0018**
Chronic kidney disease	1.48	0.34	6.38	0.5989
**Laboratory tests**				
**Calcium** (Low vs. Normal)	2.23	1.01	4.91	**0.0475**

**OR**: Odds Ratio and **CI**: Confidence Interval.

## Discussion

The present study is a retrospective cohort investigation that involved patients diagnosed with DM and hospitalized due to COVID-19. We aimed to evaluate the factors contributing to poor COVID-19 outcomes in DM patients. We have found that the mortality rate was increased with increasing age (from 5.56% in younger patients to 46% in the elderly) and with severity (from 25.71% in moderate cases to 43.77% in critical cases). We have also found that a critical severity on admission, a history of stroke, and low calcium levels on admission were significant risk factors predicting higher COVID-19 mortality in diabetic patients.

In the early COVID-19 pandemic, it has been proposed that the prevalence of diabetes in COVID-19 patients is higher than in the general population. In one study, the prevalence of DM in COVID-19 patients was 62%, which is 4.7 times higher than the general population, and the prevalence of pre-diabetes was 1.3 times higher than the general population [14].

Multiple studies during previous pandemics demonstrated that diabetic patients are at more risk for severe outcomes of infections. The rate of hospitalization was sixfold higher in patients with DM compared to healthy individuals during the H1N1 influenza pandemic [20]. Furthermore, the ICU admission rate was fourfold higher and the death rate was twofold higher [10]. During the SARS-CoV-1 pandemic in 2002, DM was associated with higher chances of complications and death [21]. In a systematic review investigating the prevalence of comorbid conditions in patients with MERS-CoV infection, it was found that DM was a predictor for severe or critical disease, and the mortality rate in the diabetic population was higher than that in the general population [22].

In early 2020, a retrospective cohort study was conducted on patients with positive COVID-19 who were admitted to 88 hospitals in the United States (US) [13]. The results of the aforementioned study concluded that the mortality rate in patients with DM (HbA1c ≥6.5%) and/or uncontrolled hyperglycemia (HbA1c <6.5% and random blood glucose ≥180 mg/dl in two or more occasions in a 24-hour period) was significantly higher than that of those without DM and/or uncontrolled hyperglycemia (28.8% vs 6.2%, *p* <0.001) [13]. Furthermore, the mortality rate was significantly higher in patients with uncontrolled hyperglycemia (41.7%) when compared to those with DM (14.8%) (*p* <0.001) [13].

In a systematic review and meta-analysis involving 9 articles, including 926 patients with positive SARS-Cov-2 infection, it was concluded that there is a significant association between DM and COVID-19-related mortality with a pooled odd ratio (OR) of 1.75 (95% CI: 1.31–2.36, P = 0.0002) [23]. In a retrospective cohort study conducted in Wuhan, China, involving 584 patients hospitalized due to a positive SARS-Cov-2 infection, it was found that DM patients have a significantly lower overall survival compared to non-DM patients [24]. In addition to that, DM (HR: 2.180, 95% CI 1.072–4.436, *p* = 0.031) and AKI (HR: 3.520, 95% CI: 1.893–6.546, *p* <0.01) were found to be the only independent risk factors for COVID-19 mortality [24].

The CORONA virus and Diabetes Outcomes (CORONADO) study is a multicentric retrospective observational study designed to compare the clinical features and outcomes of COVID-19 between age- and gender-matched diabetic and non-diabetic populations (2210 patients in each group) [25]. The result of the CORONADO study found that the composite endpoint (Invasive Mechanical Ventilation (IMV) and death), IMV alone, and death alone within 7 and 28 days of hospital admission were all significantly higher in diabetic patients [25].

As we discussed previously, we have come to the conclusion that diabetes is definitely associated with higher COVID-19-related mortality, so we designed our study to further investigate the variables that increase the mortality rate in diabetic patients hospitalized due to COVID-19. This study revealed that 38.25% of the 536 diabetic patients with severe or critical disease were deceased, while 61.75% were discharged. Additionally, the fatality rate was directly proportional to the increase in age (5.56% for the 19–40-year-old age group, 41.65% for the 41–65-year-old age category, and 46% for the older than 65-year age category). In a study involving 364 diabetic patients with severe or critical COVID-19 admitted to Renmin Hospital of Wuhan University, 59 (16.2%) patients died and 305 (83.8%) patients were discharged [26]. Furthermore, the previous study concluded that the mortality rate was increasing with age, similar to our findings [26].

This study found that the two most observed comorbidities associated with diabetic COVID-19 patients were hypertension (68.65%) and IHD (23.1%). Similar findings were observed in another study, where the prevalence of hypertension and coronary artery diseases (CADs) in diabetic patients with COVID-19 reached 61.3% and 14.5%, respectively [27]. Our study found that in patients with heart failure, stroke, and CKD, the mortality rate was significantly higher than the discharge rate.

When comparing patients in term of DM control, we found that there was no statistically significant difference in survival between well-controlled and poor-controlled DM. Raoufi et al. conducted a comparison of the clinical outcomes and imaging findings between COVID-19 patients who had well-controlled diabetes and those with poorly-controlled DM [28]. Similar to our findings, there were no significant differences in clinical outcomes (death and recovery) between patients with well-controlled and poorly-controlled DM (p > 0.05) [28]. The results of our study are consistent with the findings of the CORONADO study on hospitalized COVID-19 patients with DM in which no association was observed between HbA1c values and either mortality or mechanical ventilation and/or mortality in COVID-19 patients with DM [25]. In contrast, one study found that poorly-controlled diabetic patients have a considerably reduced chances of surviving compared to well-controlled patients (*p* = 0.001) [27]. However, 77.2% of the participants in the aforementioned study had well controlled blood glucose [27], unlike our study, in which blood glucose was well controlled in only 34.2% of patients. The close relation in the survival probability between well-controlled and poorly controlled diabetes in our study could be explained by illustrating the impact of age on mortality. The average age of patients with well-controlled diabetes was higher than that of those with poorly-controlled diabetes (66.57 vs. 63.74 years old). Moreover, of the 77 patients who died with well-controlled diabetes, 56 (72.73%) were older than 65 years old. Compared to younger patients, older individuals exhibit higher mortality rates, with a mortality rate of 4.5% for those aged over 60 in contrast to 1.4% for individuals under 60 [29]. This suggests that the mortality rate in patients with well-controlled diabetes might be increased due to the effect of increasing age.

This study found that the fatality rate of ARDS in diabetic patients hospitalized with COVID-19 was 39.63%, which is significantly lower than the discharge rate. One meta-analysis concluded that the overall fatality rate of in hospitalized COVID-19 individuals complicated with ARDS was 39%, which is similar to our finding [30]. In our hospital the definition of ARDS is acute onset of dyspnea, hypoxemia, and bilateral infiltrates on chest imaging. The significant difference between the discharge and mortality rates in diabetic patients with ARDS syndrome may be clarified by the lower age of individuals who were discharged (62 vs. 68 years old). We have also found that the mortality rate of diabetic patients complicated by acute respiratory failure was 37.35% which is significantly lower than the discharge rate. In a randomized trial by Perkins et al, it was found that the 30-day fatality rate of hospitalized COVID-19 individuals with acute respiratory failure was 36.3% and 44.4% for those who were treated with Continuous Positive Airway Pressure (CPAP) and oxygen therapy, respectively [31].

Our results suggested that a critical severity on admission, a history of stroke, and low calcium levels on admission are all risk factors indicating increased fatality in diabetic patients with COVID-19. Another study found that the severity of COVID-19 at admission (OR: 2.03, 95% CI: 1.04–3.97) and the presence of cardiovascular disease (OR: 3.84, 95% CI: 1.97–7.48) were independent risk factors for poor COVID-19 outcomes in diabetic patients [27]. In addition to that, in a meta-analysis of 2032 individuals from 7 trials, serum calcium was found to be lower in patients with a bad outcome, which is a composite of mortality and severity [32]. According to the meta-analysis conducted by Alemzadeh et al., a significant association was found between hypocalcemia and increased mortality in patients with COVID-19. Calcium levels may serve as a valuable laboratory marker of disease aggressiveness that may be rapidly assessed in emergency scenarios, aiding doctors in the identification of severe COVID-19 patients [33]. Serum levels of inflammatory markers, including cytokines, exhibit elevated concentrations in COVID-19 patients compared to those observed in healthy individuals [34]. The activity of cytokines can interfere with calcium receptors expression consequently disrupting the equilibrium of serum calcium levels [35]. The correlation between hypocalcemia, heightened infection severity, and subsequent higher mortality can be elucidated by the complex interrelationship between serum calcium levels and the intricate mechanisms of the immune system [36, 37].

One of the limitations that we faced during the conduct of this study was the absence of data regarding blood glucose levels during the hospital stay to evaluate the effect of uncontrolled hyperglycemia on the outcomes of COVID-19. Another limitation was that the data regarding lab results in some patients was not available or not performed, which may have influenced the analysis in some situations. The main strength of our study is that we have evaluated the effect of all comorbidities, lab results, and complications on the outcomes of diabetic patients hospitalized due to COVID-19.

## Conclusion

Diabetes is a common comorbidity in patients hospitalized due to the COVID-19. Poor COVID-19 prognosis and higher mortality are observed in diabetic patients infected with COVID-19. COVID-19 has several demographics, laboratory tests, and clinical risk factors, but patients with a history of stroke and/or hypocalcemia should be treated with caution. The findings of this study suggested that reduced calcium levels could potentially indicate higher mortality due to COVID-19 in patients with DM. Close observation of diabetic patients who are being treated in hospitals for COVID-19, particularly those with critical disease or a history of stroke, may improve their prognosis and reduce death.

## Supporting information

S1 Table Normal ranges of laboratory tests. (PDF)

S2 Table Insignificant laboratory test at admission for DM patients hospitalized due to COVID-19 infection. (PDF)
