# Supplementary material for: Factors contributing to poor COVID-19 outcomes in diabetic patients: Findings from a single-center cohort study
Source: PLoS One. 2023 Aug 31;18(8):e0290946. doi: 10.1371/journal.pone.0290946 (PMC10470961; doi:10.1371/journal.pone.0290946)
Supplement: S1 Table — (PDF) [file pone.0290946.s001.pdf]

S1 Table. Normal ranges of laboratory tests.

Laboratory Test	Normal Range
**pH**	7.35-7.45
**PCO2 (mmHg)**	35-45
**HCO3 (mmHg)**	22-26
**PaO2 (mmHg)**	75-100
**O2 saturation (%)**	80-100
**ESR (mm/hr)**	
Male	0-22
Female	0-29
**Ca2+ (mmol/L)**	2.13-2.55
**Mg+ (mmol/L)**	0.85-1.1
**PO4+ (mmol/L)**	0.97-1.45
**Urea (mmol/L)**	2.1-8.5
**Creatinine (μmol/L)**	
Male	65.4-119.3
Female	52.2-91.9
**Total Protein (g/L)**	60-83
**Total bilirubin (μmol/L)**	1.71-20.5
**Direct bilirubin (μmol/L)**	<5.1
**ALP (IU/L)**	44-147
**AST (IU/L)**	8-33
**GGT (IU/L)**	5-40
**INR**	<1.1
**PT (seconds)**	11-13.5
**CK (U/L)**	22-198
**CK-MB (U/L)**	5-25
**WBC (x 10^6/L)**	4500-11000
**RBC (x 10^9/L)**	
Male	4-5.9
Female	3.8-5.2
**Haemoglobin (g/dL)**	
Male	13.2-16.6
Female	11.6-15
**Haematocrit (%)**	
Male	41-50
Female	36-48
**MPV (femtoliters)**	7-9
**MCH (picograms)**	27.5-33.2
**MCHC (g/dL)**	32-36
**Lymphocytes (%)**	20-40
**Monocytes (%)**	2-8
**Neutrophils**	40-60
**Eosinophils**	1-4%
**Basophils**	0.5-1
