# Supplementary material for: Factors contributing to poor COVID-19 outcomes in diabetic patients: Findings from a single-center cohort study
Source: PLoS One. 2023 Aug 31;18(8):e0290946. doi: 10.1371/journal.pone.0290946 (PMC10470961; doi:10.1371/journal.pone.0290946)
Supplement: S2 Table — (PDF) [file pone.0290946.s002.pdf]

**S2 Table.** Insignificant laboratory test at admission for DM patients hospitalized due to COVID-19 infection.				
	**Total**	**Death (n=223)**	**Discharged (n=383)**	**p value**
**O2 saturation**				0.2482
High				
Low	175	68/175 (38.86%)	107/175 (61.14%)	
Normal	335	148/335 (44.18%)	187/335 (55.82%)	
**C-reactive Protein (CRP)**				0.0825
High	458	173/458 (37.77%)	285/458 (62.23%)	
Low	5	0/5 (0%)	5/5 (100%)	
Normal				
**Erythrocyte Sedimentation Rate (ESR)**				0.2533
High	494	178/494 (36.03%)	316/494 (63.97%)	
Low				
Normal	16	8/16 (50%)	8/16 (50%)	
**D-Dimer**				0.2958
High	506	189/506 (37.35%)	317/506 (62.65%)	
Low	3	2/3 (66.67%)	1/3 (3.33%)	
**LDH**				0.2450
High	462	169/462 (36.58%)	293/462 (63.42%)	
Low				
Normal	14	3/14 (21.43%)	11/14 (78.57%)	
**Potassium**				0.1101
High	44	22/44 (50%)	22/44 (50%)	
Low	56	24/56 (42.86%)	32/56 (57.14%)	
Normal	473	168/473 (35.52%)	305/473 (64.48%)	
**ALT**				0.8363
High	110	41/110 (37.27%)	69/110 (62.73%)	
Low				
Normal	446	171/446 (38.34%)	275/446 (61.66%)	
**ALP**				**0.0436**
High	66	33/66 (50%)	33/66 (50%)	
Low	15	3/15 (20%)	12/15 (80%)	
Normal	475	176/475 (37.05%)	299/475 (62.95%)	
**AST**				**0.0068**
High	217	98/217 (45.16%)	119/217 (54.84%)	
Low				
Normal	338	114/338 (33.73%)	224/338 (66.27%)	
**GGT**				0.3622
High	331	132/331 (39.88%)	199/331 (60.12%)	
Low				
Normal	222	80/222 (36.04%)	142/222 (63.96%)	
**Prothrombin Time (PT)**				0.1466
High	356	145/356 (40.73%)	211/356 (59.27%)	
Low				
Normal	98	32/98 (32.65%)	66/98 (67.35%)	
**Troponin T**				0.1315
High	13	7/13 (53.85%)	6/13 (46.15%)	

Low	254	85/254 (33.46%)	169/254 (66.54%)	
Normal				
**MCV**				0.5849
High	4	2/4 (50%)	2/4 (50%)	
Low	154	53/154 (34.42%)	101/154 (65.58%)	
Normal	426	164/426 (38.5%)	262/426 (61.5%)	
**MCH**				0.4174
High	8	4/8 (50%)	4/8 (50%)	
Low	182	62/182 (34.07%)	120/182 (65.93%)	
Normal	394	153/394 (38.83%)	241/394 (61.17%)	
**MCHC**				0.1982
High	8	3/8 (37.5%)	5/8 (62.5%)	
Low	37	19/37 (51.35%)	18/37 (48.65%)	
Normal	539	197/539 (36.55%)	342/539 (63.45%)	
**Platelet**				0.1879
High	57	22/57 (38.6%)	35/57 (61.4%)	
Low	85	39/85 (45.88%)	46/85 (54.12%)	
Normal	437	155/437 (35.47%)	282/437 (64.53%)	
**MPV**				0.2932
High	314	126/314 (40.13%)	188/314 (59.87%)	
Low	5	1/5 (20%)	4/5 (80%)	
Normal	265	92/265 (34.72%)	173/265 (65.28%)	
**Neutrophils**				0.1344
High	558	213/558 (38.17%)	345/558 (61.83%)	
Low	7	3/7 (42.86%)	4/7 (57.14%)	
Normal	19	3/19 (15.79%)	16/19 (84.21%)	
**Eosinophils**				0.522
High	5	2/5 (40%)	3/5 (60%)	
Low	525	200/525 (38.1%)	325/525 (61.9%)	
Normal	53	16/53 (30.19%)	37/53 (69.81%)	
